# Development and validation of a multiparametric MRI-based radiomics nomogram for the tripartite discrimination of primary benign, primary malignant, and metastatic lumbar spinal tumors

**DOI:** 10.3389/fonc.2026.1772338

**Published:** 2026-06-10

**Authors:** Canghai Shen, Shuai Yang, Xi Chen, Yongjian Feng, Yancheng Song, Jianxi Zhou, Yunchuan Sun

**Affiliations:** 1 Department of Orthopedics, Cangzhou Central Hospital, Cangzhou, Hebei, China; 2 Department of Orthopedics, Hejian Hospital of Traditional Chinese Medicine, Hejian, Hebei, China; 3 Tianjin Medical University Cancer Institute and Hospital, National Clinical Research Center for Cancer, Key Laboratory of Cancer Prevention and Therapy, Tianjin, China; 4 Tianjin’s Clinical Research Center for Cancer, Department of Thoracic Oncology, Tianjin Lung Cancer Center, Tianjin Cancer Institute & Hospital, Tianjin Medical University, Tianjin, China; 5 Department of Head, Neck and Thoracic Oncology, Cangzhou Hospital of Integrated Traditional Chinese and Western Medicine-Hebei, Cangzhou, Hebei, China

**Keywords:** differential diagnosis, lumbar spinal tumors, magnetic resonance imaging, nomogram, radiomics

## Abstract

**Objective:**

To develop and validate a multiparametric magnetic resonance imaging-based radiomics nomogram for the non-invasive preoperative differentiation of primary benign, primary malignant, and metastatic lumbar spinal tumors.

**Methodology:**

This retrospective study enrolled 100 patients with pathologically confirmed lumbar tumors. Radiomics features were extracted from T1-weighted, T2-weighted, and fat-suppressed T2-weighted sequences. A radiomics signature was constructed using a two-step feature selection method comprising minimum redundancy maximum relevance and least absolute shrinkage and selection operator regression. Clinical predictors were selected via univariate and multivariate analysis. An integrated nomogram was developed by combining the radiomics signature and independent clinical predictors within a multinomial logistic regression model. The model’s performance was evaluated regarding discrimination, calibration, and clinical utility.

**Results:**

The radiomics signature comprised 11 stable features. Five independent clinical predictors were identified. The integrated nomogram demonstrated robust discrimination, with a macro-average area under the curve of 0.887 (95% CI: 0.832–0.931) in the independent test set. The nomogram showed good calibration and provided a superior net benefit across a wide range of threshold probabilities in decision curve analysis.

**Conclusion:**

The proposed nomogram, integrating radiomic and clinical data, serves as a robust and non-invasive tool for preoperatively differentiating the three types of lumbar spinal tumors, holding significant potential to support clinical decision-making.

## 1 Introduction

The spine represents one of the most frequent sites for skeletal tumors, which are broadly categorized into primary and metastatic entities (1). Primary spinal tumors are relatively rare, constituting approximately 5% of all bone neoplasms, while metastatic lesions are exceedingly more common, affecting a significant proportion of cancer patients during their disease course (2, 3). The lumbar spine, due to its volume and biomechanical role, is a predominant site for both primary and secondary tumors (4). Accurate discrimination between primary benign, primary malignant, and metastatic lumbar tumors is of paramount clinical importance, as it directly dictates the treatment strategy—ranging from conservative management or curative resection for benign lesions to palliative decompression and systemic therapy for metastases (5, 6).

Conventional magnetic resonance imaging (MRI) serves as the cornerstone for the initial evaluation of lumbar spinal tumors, providing excellent anatomical detail for assessing bone destruction, soft tissue extension, and neural compression (7). However, the qualitative interpretation of conventional MRI often falls short in reliably differentiating tumor types due to considerable overlaps in their radiological presentations (8, 9). For instance, both aggressive primary malignancies and metastases can exhibit similar features like soft tissue masses and vertebral compression fractures. Consequently, biopsy, an invasive procedure carrying inherent risks such as bleeding, infection, and potential seeding, remains the diagnostic gold standard (10). There is, therefore, an urgent need for developing non-invasive, quantitative methods to augment diagnostic accuracy and guide clinical decision-making prior to invasive interventions.

Radiomics, an emerging field in quantitative imaging analysis, addresses this challenge by converting medical images into mineable, high-dimensional data (11). By extracting a vast array of sub-visual features related to tumor morphology, intensity statistics, and textural heterogeneity, radiomics can potentially decode the underlying pathological and biological information that is imperceptible to the human eye (12, 13). This approach has demonstrated promising results in various oncologic domains, including tumor classification, prognosis prediction, and treatment response assessment (14, 15). Nevertheless, the application of a multi-parameter MRI-based radiomics approach specifically for the tripartite discrimination (primary benign vs. primary malignant vs. metastatic) of lumbar spinal tumors remains relatively unexplored (16).

Furthermore, while radiomics models can be powerful, their “black-box” nature often limits clinical interpretability and adoption. The radiomics nomogram, which integrates radiological features with clinical risk factors, provides an elegant solution by offering a visual and intuitive tool for individualized risk prediction (18). It quantifies the contribution of each predictive variable, thereby facilitating a transparent and clinically plausible decision-making process.

Therefore, the primary objective of this study was to develop and validate a multiparametric MRI-based radiomics nomogram that synergistically combines robust imaging signatures with key clinical indicators for the non-invasive preoperative discrimination of primary benign, primary malignant, and metastatic tumors in the lumbar spine. We hypothesize that this integrated model will demonstrate superior diagnostic performance and offer substantial clinical utility, potentially serving as a valuable decision-support tool for personalized management of patients with lumbar spinal tumors.

## 2 Materials and methods

### 2.1 Patient population and study design

This retrospective study was conducted after obtaining approval from the Institutional Review Boards of all participating institutions, which waived the requirement for informed consent due to the retrospective nature of the analysis. We retrospectively screened the medical records of consecutive patients who presented to our department with lumbar spinal lesions and underwent preoperative multi-parameter MRI between January 2020 and December 2023.

The inclusion criteria were as follows: (1) availability of complete preoperative T1-weighted, T2-weighted, and fat-suppressed T2-weighted (FS-T2WI) MRI sequences; (2) a confirmed pathological diagnosis of the lumbar lesion obtained via surgery or biopsy, which served as the reference standard; (3) no history of any anti-tumor therapy (e.g., radiotherapy, chemotherapy, or ablation) prior to the MRI examination.

The exclusion criteria were: (1) poor image quality that precluded accurate segmentation (e.g., significant artifacts or signal-to-noise ratio ≤ 1.0); (2) incomplete clinical or laboratory data required for the analysis; (3) lesions that were ultimately confirmed to be non-neoplastic (e.g., infectious spondylitis or traumatic fractures).

Based on the pathological results, the enrolled patients were categorized into three groups: the primary benign tumor group, the primary malignant tumor group, and the metastatic tumor group.

A total of 100 patients met all the criteria and constituted the final study cohort.

To ensure the generalizability of the model and avoid overfitting, the entire cohort was randomly split into a training set and an independent test set at a ratio of 7:3 using a stratified sampling method. This stratification was performed based on the pathological tumor type (primary benign, primary malignant, metastatic) to maintain the same class distribution in both subsets. The splitting process was implemented using the createDataPartition function in the caret package of R software, with a fixed random seed (seed = 12345) to ensure full reproducibility. The training set (n=70) was exclusively used for feature selection, model training, and hyperparameter optimization, while the independent test set (n=30) was completely held out and only used for the final, unbiased evaluation of the model’s performance. No data from the test set was used at any stage of model development.

### 2.2 Image acquisition and preprocessing

All preoperative MRI examinations were performed using a 3.0T MR scanner (Discovery MR750, GE Healthcare, USA) with a dedicated spinal coil to ensure high-quality image acquisition. The standardized imaging protocol included sagittal T1-weighted imaging (T1WI), T2-weighted imaging (T2WI), and fat-suppressed T2-weighted imaging (FS-T2WI), which are routinely used in clinical practice for spinal evaluation. The detailed parameters for each sequence are summarized in **Table 1**. To minimize the influence of non-biological variations on feature extraction, all acquired images underwent a standardized preprocessing pipeline using the 3D Slicer software (version 4.13.0) and its integrated modules. First, the N4ITK bias field correction algorithm was applied to correct for intensity inhomogeneity caused by magnetic field imperfections, with the iteration parameter set to 50. Subsequently, all images were resampled to an isotropic voxel size of 1.5×1.5×1.5 mm³ using B-spline interpolation. This step standardizes the spatial resolution across all patients, ensuring that the extracted morphological features are comparable and not influenced by varying voxel dimensions. Finally, Z-score normalization was performed on the image intensity values within the segmented tumor volume of interest (VOI) to mitigate variations in signal intensity arising from different scanner calibrations or gain settings.

**Table 1:** Standardized pipeline and parameters for radiomics feature extraction.

Procedure step	Detailed specifications
MRI Scanner and Sequence Parameters	Scanner: GE Discovery MR750 3.0T MRI System Sequence Parameters (clinical adjustment ±50ms): • T1WI: TR 550–750 ms, TE 15–25 ms, Matrix 240×320, Slice Thickness 4.0 mm, Slice Gap 0.8 mm (Scan Time 3–4 min) • T2WI: TR 2800–3200 ms, TE 105–115 ms, Matrix 288×384 • FS-T2WI: TR 3200–3800 ms, TE 60–100 ms, Matrix 384×384
Image Preprocessing	Software: 3D-Slicer (v4.13.0) Steps: ① N4 Bias Field Correction (50 iterations; grayscale standard deviation reduction ≥30%) ② Voxel Resampling (Isotropic 1.5×1.5×1.5 mm³) ③ Intensity Normalization (Z-score Standardization)
ROI Segmentation	Software: ITK-SNAP (v3.8.0) Criteria: ① Segmentation of the solid tumor region only (excluding necrosis and cystic components) ② Performed independently by two attending radiologists (with 5 and 7 years of experience) + arbitration by a senior associate chief physician (10 years of experience) ③ Repeated segmentation with a 2-week interval
Feature Extraction	Software/Tool: PyRadiomics (v3.0.1) within 3D-Slicer Features per sequence: • Morphological: 12 • First-order Statistics: 20 • Textural: 45 • Wavelet-based: 32 Total per sequence: 109 features

MRI, Magnetic Resonance Imaging; T1WI, T1-weighted imaging; T2WI, T2-weighted imaging; FS-T2WI, Fat-Suppressed T2-weighted imaging; TR, Repetition Time; TE, Echo Time; ROI, Region of Interest.

### 2.3 Tumor segmentation and radiomics feature extraction

The workflow for tumor segmentation and feature extraction was conducted in a standardized manner to ensure the robustness and reproducibility of the extracted features.

#### 2.3.1 Volumetric segmentation of tumor

The volumetric regions of interest (VOIs) encompassing the entire tumorous solid region were manually delineated on each slice of the sagittal T1WI, T2WI, and FS-T2WI sequences using the open-source software ITK-SNAP (version 3.8.0; www.itk-snap.org). To ensure clinical relevance and minimize confounding signals, the segmentation adhered to the following criteria: (1) only the solid tumor component was outlined, carefully excluding areas of obvious necrosis, cystic changes, hemorrhage, and surrounding edema; (2) the segmentation was performed independently by two radiologists (with 5 and 7 years of experience in musculoskeletal imaging, respectively), who were blinded to the pathological diagnosis and clinical grouping; (3) in cases of significant disagreement between the two initial readers, a third senior radiologist (with over 10 years of experience) served as an arbiter to make the final decision.

#### 2.3.2 Radiomics feature extraction

Following the image preprocessing and finalized segmentation, radiomics features were extracted from the VOI of each MRI sequence (T1WI, T2WI, FS-T2WI) for every patient. The extraction was performed using the PyRadiomics python package (v3.0.1) integrated within 3D Slicer, which complies with the Image Biomarker Standardization Initiative (IBSI) (19). A comprehensive set of 109 features was extracted from each VOI, which were categorized into the following classes: (1) Shape-based features (n=12): describing the three-dimensional size and geometry of the tumor (e.g., volume, sphericity, surface area); (2) First-order statistics features (n=20): describing the distribution of voxel intensities within the VOI (e.g., mean, median, kurtosis, skewness, entropy); (3) Texture features (n=45): quantifying the intra-tumoral heterogeneity and spatial relationships between voxels. These were derived from Gray Level Co-occurrence Matrix (GLCM), Gray Level Run Length Matrix (GLRLM), Gray Level Size Zone Matrix (GLSZM), and Neighboring Gray Tone Difference Matrix (NGTDM); (4) Wavelet features (n=32): generated by applying wavelet filters to the original image, resulting in decomposed images (e.g., LHL, HLL, where L=Low, H=High pass filtering) from which first-order and texture features were subsequently extracted. This yielded an initial feature pool of 327 features per patient (109 features/sequence × 3 sequences).

#### 2.3.3 Assessment of segmentation consistency

To quantitatively evaluate the reproducibility of the manual segmentations, both intra- and inter-observer reliability were assessed using the Intraclass Correlation Coefficient (ICC). One radiologist repeated the segmentation process for 30 randomly selected cases after a 2-week interval to calculate intra-observer ICC. The segmentations from the two independent radiologists were compared to calculate inter-observer ICC. Features with an ICC value greater than 0.85 in both assessments were considered to have excellent consistency and were retained for subsequent analysis, as detailed in **Table 2**.

**Table 2:** Intra- and inter-observer reproducibility of tumor segmentation (ICC, 95% CI).

MRI sequence	Feature category	Intra-observer ICC (rater 1, two sessions)	Inter-observer ICC (rater 1 vs. rater 2)	Discrepancy resolution	Consistency assessment
T1WI	Morphological	0.952 (0.901-0.978)	0.938 (0.885-0.967)	2 cases with >10% discrepancy; ICC improved to 0.945 after arbitration	Excellent (≥0.85)
	First-order Statistics	0.926 (0.868-0.959)	0.905 (0.841-0.943)	Consistency improved for 1 case after reviewing axial images	Excellent
	Textural	0.893 (0.821-0.937)	0.876 (0.795-0.924)	2 noise-sensitive features excluded	Excellent
	Wavelet-based	0.887 (0.813-0.932)	0.862 (0.778-0.918)	No specific action	Excellent
T2WI	Morphological	0.961 (0.918-0.982)	0.945 (0.897-0.972)	No discrepant cases	Excellent
	First-order Statistics	0.935 (0.879-0.965)	0.912 (0.850-0.948)	ICC improved to 0.920 after normalization in 1 case	Excellent
	Textural	0.901 (0.835-0.941)	0.884 (0.806-0.929)	Excluded 1 feature: ‘Gray Level Run Length Non-Uniformity’	Excellent
	Wavelet-based	0.895 (0.825-0.938)	0.871 (0.789-0.921)	No specific action	Excellent
FS-T2WI	Morphological	0.968 (0.929-0.985)	0.953 (0.909-0.976)	No discrepant cases	Excellent
	First-order Statistics	0.942 (0.888-0.969)	0.921 (0.862-0.953)	No discrepant cases	Excellent
	Textural	0.915 (0.852-0.949)	0.897 (0.823-0.939)	No specific action	Excellent
	Wavelet-based	0.903 (0.838-0.942)	0.889 (0.812-0.932)	No specific action	Excellent

ICC, Intraclass Correlation Coefficient; CI, Confidence Interval; T1WI, T1-weighted imaging; T2WI, T2-weighted imaging; FS-T2WI, Fat-Suppressed T2-weighted imaging.

All ICC values were ≥ 0.85, indicating excellent segmentation reproducibility.

### 2.4 Feature selection and radiomics signature construction

Given the high-dimensional nature of the initial radiomics feature pool relative to the sample size, a series of measures were implemented to mitigate overfitting risk. A two-step feature selection strategy was employed to identify a stable, non-redundant feature set for model construction.

#### 2.4.1 Minimum redundancy maximum relevance filtering

The first step involved the Minimum Redundancy Maximum Relevance (mRMR) algorithm, which aims to select features that have the highest relevance to the outcome labels (tumor types) while maintaining minimal pairwise redundancy among themselves. This method effectively reduces feature dimensionality by eliminating redundant information, thereby enhancing the generalizability of the subsequent model. Following mRMR filtering, 97 features with the highest mRMR scores were retained from the initial pool of 327 for further analysis.

#### 2.4.2 Least absolute shrinkage and selection operator regression

Subsequently, the Least Absolute Shrinkage and Selection Operator (LASSO) regression method, which is particularly suited for high-dimensional data, was applied to the 97 features retained from the mRMR step. LASSO penalizes the absolute size of the regression coefficients, effectively shrinking the coefficients of less contributory features to zero and performing an embedded feature selection. A 10-fold stratified cross-validation was performed to determine the optimal value of the penalty parameter (λ) that minimized the multinomial deviance. The optimal λ value was selected via the one-standard-error (1-SE) criterion to obtain a more parsimonious and generalizable model. All LASSO calculations were performed using the glmnet package (version 4.1-8) in R software, with a fixed random seed (seed = 12345) to ensure full reproducibility. Features with non-zero coefficients at the optimal λ value were selected as the final core radiomics features, as documented in **Table 3**.

**Table 3:** Radiomics feature selection results based on the mRMR and LASSO algorithm.

MRI sequence	Initial feature count	Features after mRMR	Features after LASSO	Final selected core features (clinical/radiological significance)
T1WI	109	31	3	1. Morphological: Tumor Surface Area/Volume Ratio (reflecting invasiveness and border irregularity) 2. First-order: Histogram Skewness (reflecting asymmetry in T1WI low-intensity distribution) 3. Textural: GLCM Entropy (indicating intra-tumoral intensity heterogeneity)
T2WI	109	34	4	1. Morphological: Tumor Major Axis/Minor Axis Ratio (suggesting growth pattern and directionality) 2. First-order: 75th Percentile (associated with necrotic components) 3. Textural: GLRLM Short-Run Low Gray-Level Emphasis (differentiating solid tumor vs. edema) 4. Wavelet: db4 Low-Frequency Kurtosis (reflecting overall gray-level distribution trends)
FS-T2WI	109	32	4	1. First-order: Gray-Level Non-Uniformity (discriminating tumor parenchyma vs. edema) 2. Textural: GLSZM Small Area High Gray-Level Emphasis (suggesting multifocality in metastases) 3. Wavelet: db4 Mid-Frequency Entropy (capturing subtle structural differences) 4. Textural: GLCM Inverse Difference Moment (indicating local gray-level homogeneity)
Total	327	97	11	Morphological: 2, First-order: 3, Textural: 4, Wavelet-based: 2

mRMR, Minimum Redundancy Maximum Relevance; LASSO, Least Absolute Shrinkage and Selection Operator; GLCM, Gray Level Co-occurrence Matrix; GLRLM, Gray Level Run Length Matrix; GLSZM, Gray Level Size Zone Matrix.

#### 2.4.3 Construction of the radiomics signature

The selected 11 features were then linearly combined, weighted by their respective LASSO regression coefficients, to generate a quantitative Radiomics Signature (Rad-score) for each patient. This Rad-score represents a single, integrated value that encapsulates the discriminative information derived from the multi-parameter MRI radiomics analysis.

### 2.5 Clinical variable selection and integrated model construction

To develop a comprehensive predictive tool, we integrated the radiomics signature with clinically relevant variables through a systematic modeling process.

#### 2.5.1 Selection of clinical predictors

Clinical variables collected from the baseline data were rigorously assessed for their independent predictive value. Initially, all clinical parameters that showed a significant difference (P < 0.05) in the univariate analysis (as presented in **Table 4**) were considered as candidate variables. These included VAS pain score, neurological compression symptoms, serum tumor marker status, serum calcium, serum ALP, the presence of a soft tissue mass on MRI, the presence of a vertebral compression fracture on MRI, and the time interval from symptom onset to MRI examination.

**Table 4:** Comparison of baseline characteristics of patients with lumbar spinal tumors.

Characteristic	Primary benign group (n=30)	Primary malignant group (n=22)	Metastatic group (n=48)	Statistic	P value
Demographics and Symptoms					
Age (years)	47.8 ± 10.5	58.6 ± 9.8	55.2 ± 10.3	F=3.124	0.048
Gender, n (%)				χ²=0.066	0.968
Male	16 (53.3)	12 (54.5)	27 (56.3)		
Female	14 (46.7)	10 (45.5)	21 (43.7)		
BMI (kg/m²)	23.6 ± 2.2	22.9 ± 2.3	23.2 ± 2.1	F=0.516	0.598
Smoking History, n (%)				χ²=0.631	0.729
No	22 (73.3)	15 (68.2)	37 (77.1)		
Yes	8 (26.7)	7 (31.8)	11 (22.9)		
ECOG Score, n (%)				χ²=2.955	0.228
0-1	28 (93.3)	17 (77.3)	39 (81.3)		
2	2 (6.7)	5 (22.7)	9 (18.7)		
VAS Pain Score	3.5 (2.0, 5.5)	6.5 (4.5, 8.0)	7.0 (5.5, 8.5)	H=21.546	<0.001
Signs and Serology					
Neurological Compression Symptoms, n (%)				χ²=29.465	<0.001
Present	3 (10.0)	12 (54.5)	35 (72.9)		
Absent	27 (90.0)	10 (45.5)	13 (27.1)		
Comorbidities, n (%)				χ²=0.409	0.999
None	18 (60.0)	12 (54.5)	28 (58.3)		
Hypertension	7 (23.3)	5 (22.7)	11 (22.9)		
Diabetes Mellitus	3 (10.0)	3 (13.6)	6 (12.5)		
Hypertension + Diabetes	2 (6.7)	2 (9.2)	3 (6.3)		
Serum Tumor Markers, n (%)				χ²=41.442	<0.001
Negative	28 (93.3)	12 (54.5)	9 (18.8)		
Positive	2 (6.7)	10 (45.5)	39 (81.2)		
Serum Calcium (mmol/L)	2.3 ± 0.1	2.5 ± 0.2	2.8 ± 0.3	F=42.158	<0.001
Serum Alkaline Phosphatase (ALP, U/L)	85 ± 12	120 ± 18	150 ± 25	F=58.326	<0.001
MRI Characteristics					
Vertebral Level Involved, n (%)				χ²=0.119	0.998
L1-L2	5 (16.7)	3 (13.6)	8 (16.7)		
L3-L4	12 (40.0)	9 (40.9)	19 (39.6)		
L5-S1	13 (43.3)	10 (45.5)	21 (43.7)		
Time from Symptom Onset to MRI (months)	3.9 ± 1.4	2.8 ± 1.1	2.4 ± 1.0	F=4.268	0.017
MRI with Soft Tissue Mass, n (%)				χ²=50.780	<0.001
Yes	0 (0.0)	15 (68.2)	40 (83.3)		
No	30 (100.0)	7 (31.8)	8 (16.7)		
MRI with Vertebral Compression Fracture, n (%)				χ²=26.997	<0.001
Yes	2 (6.7)	10 (45.5)	32 (66.7)		
No	28 (93.3)	12 (54.5)	16 (33.3)		
Pathological Type, n (%)				-	-
Osteoid Osteoma	10 (33.3)	0 (0.0)	0 (0.0)		
Osteochondroma	8 (26.7)	0 (0.0)	0 (0.0)		
Vertebral Hemangioma	12 (40.0)	0 (0.0)	0 (0.0)		
Multiple Myeloma	0 (0.0)	8 (36.4)	0 (0.0)		
Spinal Osteosarcoma	0 (0.0)	7 (31.8)	0 (0.0)		
Chondrosarcoma	0 (0.0)	7 (31.8)	0 (0.0)		
Lung Cancer Metastasis	0 (0.0)	0 (0.0)	18 (37.5)		
Breast Cancer Metastasis	0 (0.0)	0 (0.0)	15 (31.2)		
Thyroid Cancer Metastasis	0 (0.0)	0 (0.0)	8 (16.7)		
Renal Cancer Metastasis	0 (0.0)	0 (0.0)	7 (14.6)		

BMI, Body Mass Index; ECOG, Eastern Cooperative Oncology Group; VAS, Visual Analogue Scale; ALP, Alkaline Phosphatase; MRI, Magnetic Resonance Imaging.

Data are presented as Mean ± Standard Deviation, Median (Q1, Q3), or Number (%). *P* values indicate statistical significance (P < 0.05). ANOVA was used for normally distributed continuous variables, the Kruskal-Wallis H test for non-normally distributed continuous variables, and the Chi-squared test for categorical variables.

Multicollinearity among these candidate variables was assessed using the Variance Inflation Factor (VIF). A VIF value exceeding 5 was considered indicative of significant collinearity. Variables with VIF > 5 were subjected to centering or were considered for exclusion. Subsequently, these clinically relevant variables were entered into a multivariable logistic regression model (with the primary benign group as the reference category) to identify independent predictors. The final set of clinical predictors was selected based on both statistical significance (P < 0.05) in the multivariable model and clinical plausibility, as detailed in **Table 5**.

**Table 5:** Selection process of clinical variables for model construction based on **Table 4**.

Clinical variables from Table 1 with P<0.05	Rationale for association with tumor type (clinical significance)	Multicollinearity (VIF value)	Included in final model?	Reason for inclusion/exclusion
1. VAS Pain Score (points)	Pain Level: Metastatic > Primary Malignant > Primary Benign, directly reflecting tumor aggressiveness (malignant tumors more significantly irritate nerves).	1.23 (VIF <2, No collinearity)	Yes	Easily obtainable (routine admission assessment) and strongly correlates with malignancy.
2. Neurological Compression Symptoms (Present/Absent)	Incidence: Metastatic > Primary Malignant > Primary Benign, as malignant tumors readily form soft tissue masses/bony destruction compressing nerves.	1.35 (No collinearity)	No	Not statistically significant in multivariate analysis (P = 0.562, **Table 6**), therefore excluded.
3. Serum Tumor Markers (Positive/Negative)	Positivity Rate: Metastatic (81.2%) > Primary Malignant (45.5%) > Primary Benign (6.7%), directly indicative of metastatic disease (often with a known primary cancer).	1.18 (No collinearity)	Yes	A core laboratory discriminative indicator, showing the most significant difference in **Table 4** (χ²=41.442).
4. Serum Calcium (mmol/L)	Level: Metastatic > Primary Malignant > Primary Benign, due to bone metabolism disruption by malignant tumors causing hypercalcemia.	1.87 (No collinearity)	Yes	Complements the identification of marker-negative metastases (e.g., some renal cell metastases only present with elevated calcium).
5. Serum ALP (U/L)	Level: Metastatic > Primary Malignant > Primary Benign, reflecting bone repair activity (stimulated by malignant tumors).	2.91 (VIF≈3, Mild collinearity)	Yes (After adjustment)	Despite mild collinearity with calcium, addressed by “centering processing”; crucial for identifying osteoblastic metastases.
6. MRI Soft Tissue Mass (Present/Absent)	Incidence: Metastatic (83.3%) > Primary Malignant (68.2%) > Primary Benign (0%), a strong indicator for benign vs. malignant differentiation.	1.42 (No collinearity)	Yes	An intuitive imaging feature, highly consistent with clinical diagnostic reasoning.
7. MRI Vertebral Compression Fracture (Present/Absent)	Incidence: Metastatic (66.7%) > Primary Malignant (45.5%) > Primary Benign (6.7%), reflects tumor-induced compromise of vertebral load-bearing capacity.	1.56 (No collinearity)	No	Not statistically significant in multivariate analysis (P = 0.896, **Table 6**), therefore excluded.
8. Time from Symptom Onset to MRI (months)	Duration: Metastatic (2.4 mo) < Primary Malignant (2.8 mo) < Primary Benign (3.9 mo), as metastases progress faster.	1.27 (No collinearity)	No	Low clinical practicality (patient recall of symptom onset is often inaccurate) and low discriminative contribution (F = 4.268).

VAS, Visual Analogue Scale; ALP, Alkaline Phosphatase; MRI, Magnetic Resonance Imaging; VIF, Variance Inflation Factor.

VIF > 5 was considered indicative of significant multicollinearity. Centering processing was applied to variables with VIF ≈ 3 to mitigate mild collinearity.

#### 2.5.2 Construction of the integrated radiomics-nomogram

**Figure 1:**
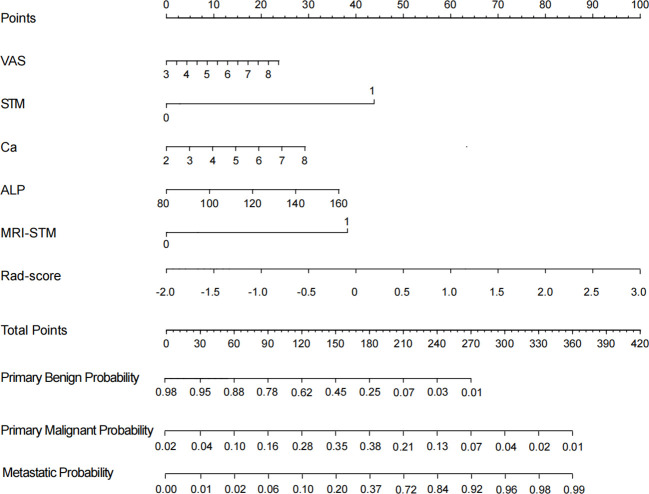
The multiparametric MRI-based radiomics nomogram for three-class tumor differentiation. The nomogram was constructed using the training set (n=70) by integrating five clinical predictors (VAS, visual analog scale pain score; STM, serum tumor markers; Ca, serum calcium; ALP, serum alkaline phosphatase; MRI-STM, MRI soft tissue mass) and the radiomics signature (Rad-score, derived from 11 core features). For clinical use: Locate each predictor value on its axis, sum the corresponding points, and read the predicted probabilities for the three tumor types from the probability axes. The tumor type with the highest probability is the model’s diagnosis. A detailed 5-step procedure is provided in Section 2.5.2. The highest probability of primary malignant tumors occurs within the 150–180 total points interval. A detailed probability lookup table for this range is available in **Supplementary Table 2**.

The final integrated model was formulated as a multinomial logistic regression model, which is specifically designed for three-class classification tasks. Unlike binary logistic regression that produces a single probability, this model generates three mutually exclusive and collectively exhaustive probabilities for each patient, corresponding to the likelihood of the tumor being primary benign, primary malignant, or metastatic, respectively. The sum of these three probabilities equals 1. The predictors included the Rad-score (a single integrated value derived from the linear combination of the 11 selected radiomics features weighted by their LASSO coefficients) and the five independent clinical predictors identified in the previous step. This combined model was then visually presented as a radiomics nomogram using the rms package (version 6.7-1) in R software, with the maximum scale of each predictor axis set to 100 points for standardized scoring (**Figure 1**). All nomogram construction steps were performed with a fixed random seed (seed = 12345) to ensure reproducibility. The nomogram consists of three main components: (1) a predictor axis for each independent variable, with corresponding point values; (2) a total points axis; and (3) three separate probability axes, one for each tumor type. This structure allows clinicians to quantify the individual contribution of each predictor and calculate the specific predicted probability for each of the three tumor categories for any individual patient.

The standardized procedure for using the nomogram is as follows: (1) Locate the patient’s value for each predictor (VAS pain score, serum tumor marker status, serum calcium, serum ALP, presence of MRI soft tissue mass, and Rad-score) on its corresponding axis; (2) Draw a vertical line upward from each value to the top “Points” axis to determine the score for that predictor; (3) Sum the scores of all six predictors to obtain the total points; (4) Draw a vertical line downward from the total points axis to each of the three probability axes; (5) Read the predicted probability for primary benign, primary malignant, and metastatic tumors respectively. The tumor type with the highest predicted probability is considered the model’s diagnostic result.

#### 2.5.3 Model performance and validation

**Figure 2:**
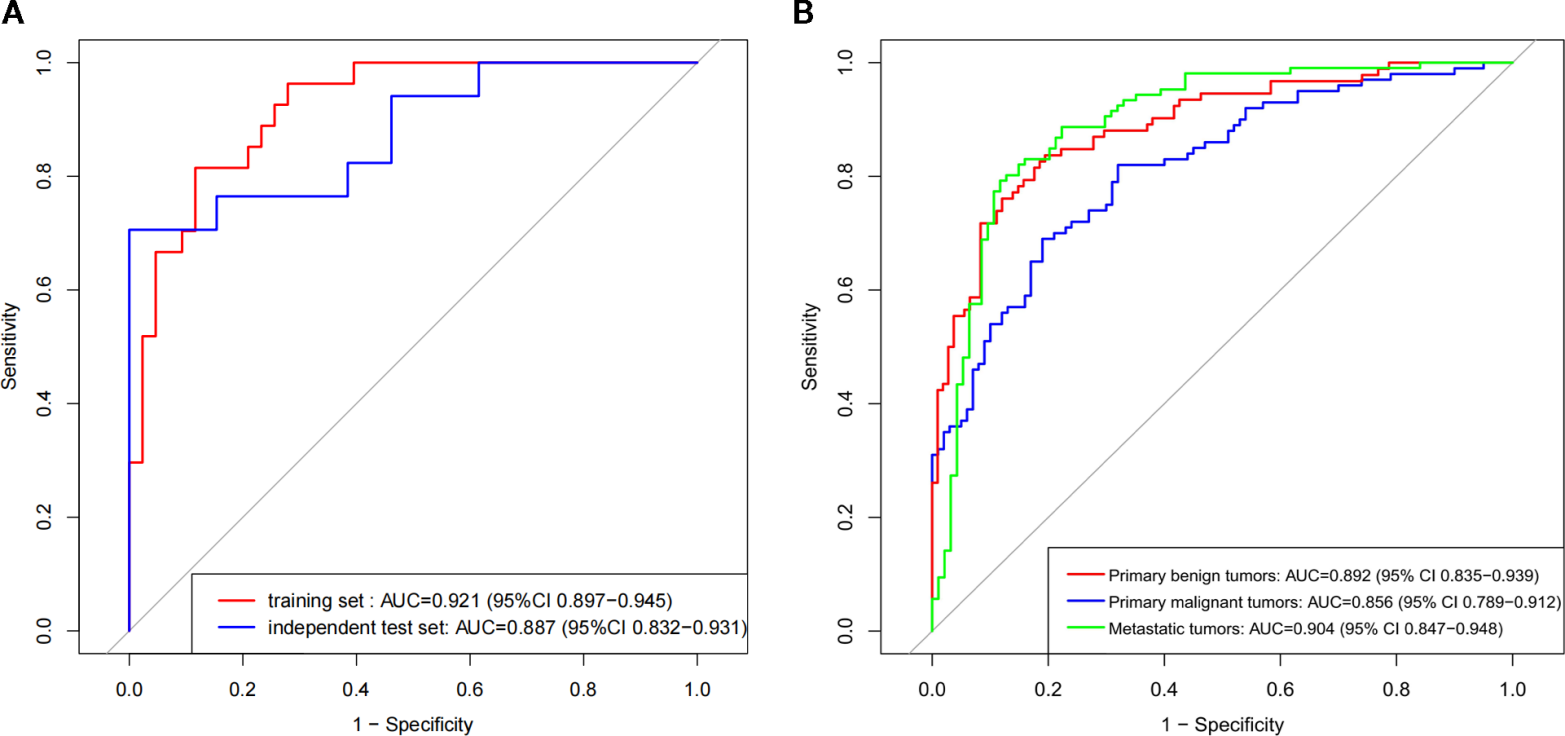
Receiver operating characteristic (ROC) curve of the integrated nomogram for three-class discrimination. **(A)** Macro-average ROC curves comparing overall model performance between the training set and the independent test set. The upper curve represents the training set (n=70), with an AUC of 0.921 (95% CI: 0.897–0.945). The lower curve represents the independent test set (n=30), which was not used in any model development step, with an AUC of 0.887 (95% CI: 0.832–0.931). **(B)** One-vs-rest ROC curves for individual tumor type identification in the independent test set. The three curves correspond to primary benign vs. other types (AUC = 0.892, 95% CI: 0.835–0.939), primary malignant vs. other types (AUC = 0.856, 95% CI: 0.789–0.912), and metastatic vs. other types (AUC = 0.904, 95% CI: 0.847–0.948), respectively.

**Figure 3:**
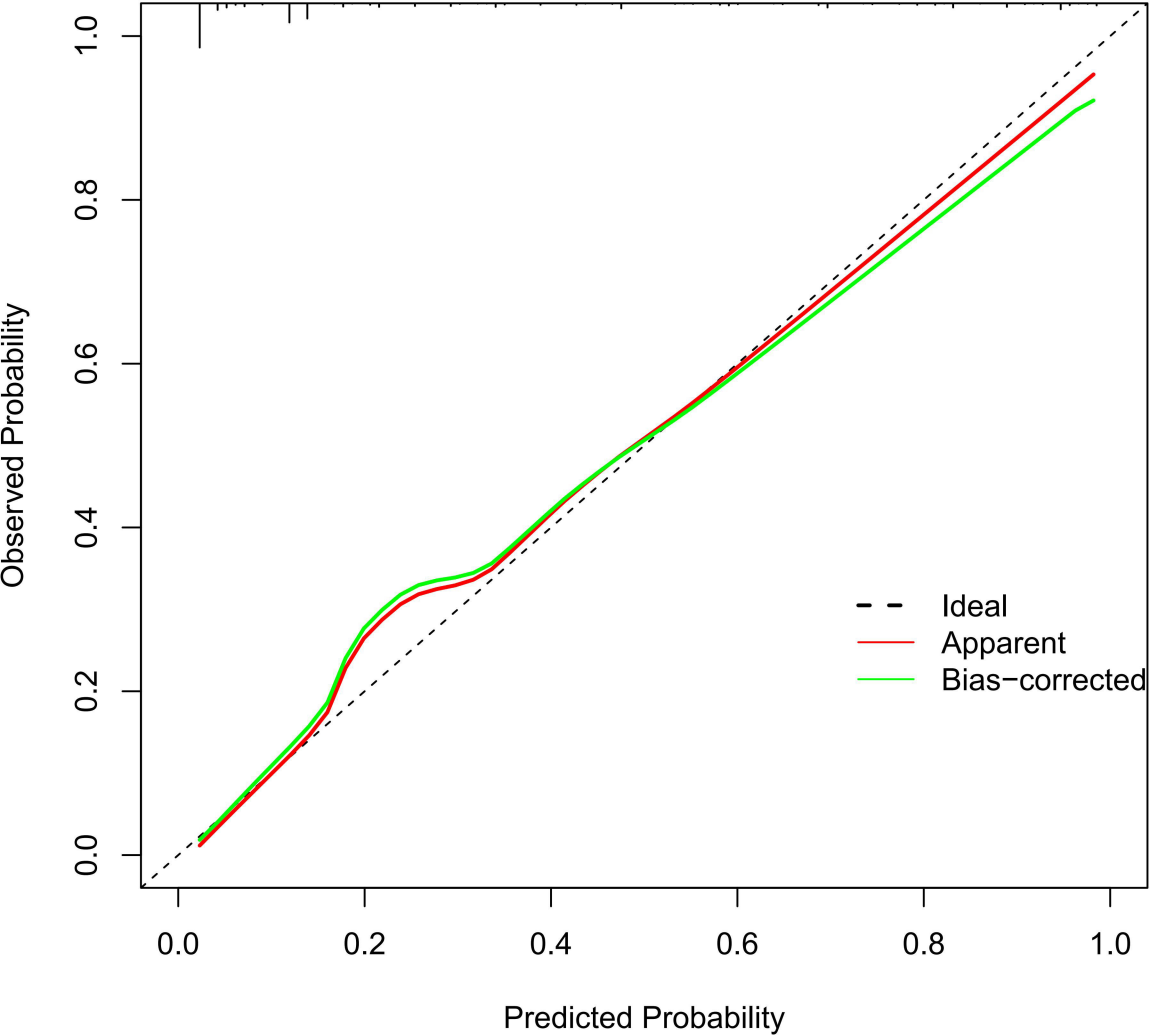
Calibration curves of the nomogram in the independent test set (n=30). The plot assesses the agreement between the predicted probabilities (x-axis) and the actual observed frequencies (y-axis) for the three tumor classifications. The diagonal solid line represents the ideal perfect calibration. The ‘Apparent’ (apparent performance) and ‘Bias-corrected’ (1000-bootstrap resampling corrected performance) curves closely align with the ideal line, indicating good calibration. The Hosmer-Lemeshow test yielded a χ² value of 7.213 and a P-value of 0.407, confirming no significant deviation from perfect fit.

The performance of the integrated nomogram was evaluated in terms of discrimination, calibration, and clinical utility. Discrimination: The model’s ability to differentiate between the three tumor types was assessed using receiver operating characteristic (ROC) curves and calculation of the area under the curve (AUC). Three types of AUC were reported: (1) macro-average AUC, representing overall three-class classification performance; (2) one-vs-rest AUC for each individual tumor type, representing the model’s ability to identify a specific tumor type from the other two; and (3) pairwise AUC for each combination of two tumor types, representing the model’s ability to distinguish between two specific tumor types (**Figure 2**). Calibration: The agreement between predicted probabilities and actual observed outcomes was evaluated using calibration curves and the Hosmer-Lemeshow goodness-of-fit test (**Figure 3**). Clinical Utility: Decision Curve Analysis (DCA) was performed to quantify the standardized net benefit (SNB) across a continuum of threshold probabilities. The SNB was calculated using the standard equation: SNB = [TP - (FP × p)/(1 - p)]/N, where TP = true positive predictions, FP = false positive predictions, p = threshold probability, and N = total number of patients. For this three-class classification problem, we adopted the widely accepted one-vs-rest (OvR) decision curve analysis strategy. Specifically, we evaluated the clinical utility separately for each tumor type by treating that type as the “target positive class” and the other two types combined as the “negative class”. In this framework:

True Positive (TP) for class X: Number of patients with actual pathological diagnosis of class X whose predicted probability of class X was ≥ threshold p.

False Positive (FP) for class X: Number of patients with actual pathological diagnosis not of class X whose predicted probability of class X was ≥ threshold p.

Threshold p: The minimum probability at which a clinician would consider initiating further diagnostic or therapeutic interventions specific to that tumor type.

Threshold probabilities ranged from 0.05 to 0.95, covering the full clinically relevant spectrum. The SNB of the nomogram was compared against two default strategies: treat all patients and treat no patients.

### 2.6 Statistical analysis

All statistical analyses were performed using R software (version 4.2.2; R Foundation for Statistical Computing) and Python (version 3.9). A two-sided P-value of less than 0.05 was considered statistically significant for all tests, unless otherwise specified.

#### 2.6.1 Descriptive statistics and baseline comparisons

Normally distributed continuous variables were presented as mean ± standard deviation and compared among the three groups using one-way analysis of variance (ANOVA), followed by *post-hoc* Tukey tests for pairwise comparisons. Non-normally distributed continuous variables were expressed as median with interquartile range (IQR) and compared using the Kruskal-Wallis H test, with *post-hoc* Dunn’s tests. Categorical variables were summarized as frequencies and percentages and compared using the Chi-squared test or Fisher’s exact test, as appropriate. These results are comprehensively presented in **Table 4**.

#### 2.6.2 Analysis specific to model development and evaluation

As detailed in preceding sections, specific statistical methods were employed for key steps in the model development pipeline: The Intraclass Correlation Coefficient (ICC) was used for assessing feature stability (2.3). The mRMR and LASSO algorithms were used for feature selection (2.4). Multivariable logistic regression was used for final model construction (2.5).

#### 2.6.3 Model performance assessment

The discriminatory performance of the final nomogram was evaluated using the Area Under the Receiver Operating Characteristic Curve (AUC), with DeLong’s test used if needed for comparing AUCs. Calibration was assessed visually with calibration plots and statistically with the Hosmer-Lemeshow goodness-of-fit test. Clinical utility was quantified using Decision Curve Analysis (DCA).

## 3 Results

### 3.1 Patient characteristics and cohort description

A total of 100 patients with pathologically confirmed lumbar spinal tumors were enrolled and categorized into three groups: primary benign (n=30), primary malignant (n=22), and metastatic (n=48). Comparative analysis of baseline characteristics revealed statistically significant differences in 9 parameters among the three groups (**Table 4**).

The cohort was randomly split into a training set (n=70) and an independent test set (n=30) as described in Section 2.1. No statistically significant differences were observed in any demographic, clinical, or imaging parameters between the two subsets (all P > 0.05, **Supplementary Table 1**), confirming the validity of the cohort split.

### 3.2 Radiomics feature extraction, stability, and selection

#### 3.2.1 Segmentation reproducibility, feature extraction, and standardization pipeline

To ensure feature robustness, inter- and intra-observer reproducibility of tumor VOI segmentation was assessed using the intraclass correlation coefficient (ICC) as described in Section 2.3.3. All core features across all MRI sequences demonstrated excellent reproducibility, with ICCs ≥ 0.85 (**Table 2**). FS-T2WI exhibited the highest consistency for morphological features (inter-observer ICC = 0.953). Minor initial segmentation discrepancies (n=6) were resolved by a senior arbiter.

Radiomics feature extraction followed a standardized pipeline detailed in **Table 1**. A total of 109 features per sequence (morphological, first-order statistical, textural, and wavelet) were extracted using the PyRadiomics package (v3.0.1), yielding an initial feature pool of 327 features per patient.

#### 3.2.2 Selection of core radiomics signature

Following the two-step feature selection pipeline described in Section 2.4, 11 core radiomics features were ultimately selected from the initial pool of 327 (**Table 3**). These included 2 morphological, 3 first-order, 4 textural, and 2 wavelet features. FS-T2WI contributed the most features (n=4, 36.4%), underscoring its high discriminatory value. The selected features demonstrated strong biological plausibility: for example, the “Surface Area/Volume Ratio” from T1WI reflects invasive border irregularity, while “Gray-Level Non-Uniformity” from FS-T2WI indicates intra-tumoral heterogeneity characteristic of metastases.

### 3.3 Predictive model construction

#### 3.3.1 Clinical variable selection

Eight clinical variables with P < 0.05 in univariate analysis were initially considered. Variance inflation factor (VIF) analysis confirmed the absence of significant multicollinearity (all VIFs < 2 after centering). Following multivariate logistic regression analysis, five independent clinical predictors were retained for the final model: VAS pain score, serum tumor marker positivity, serum calcium level, serum ALP level, and the presence of an MRI soft tissue mass (**Table 5**).

#### 3.3.2 Integrated radiomics-clinicopathological model

A multinomial logistic regression model was constructed. The predictors included the 11-feature radiomics signature (represented by a single integrated Rad-score) and the five independent clinical variables identified in the multivariate analysis (**Table 5**). **Table 6** presents the regression coefficients, Odds Ratios (OR), and P-values for all candidate clinical and radiomics variables evaluated in the model. Variables with P < 0.05 in the multivariate analysis were retained as the final core predictors, which included all five clinical variables and the integrated Rad-score (derived from 11 radiomics features).

**Table 6:** Coefficients of all candidate variables in the multinomial logistic regression model (reference category: primary benign group).

Variable category	Variable	Regression coefficient (β)	Standard error (SE)	Odds ratio (OR) (95% CI)	P value
Clinical Variables	VAS Pain Score (per 1-point increase)	0.826	0.218	2.385 (1.097-3.748)	0.006
	Neurological Compression Symptoms (Present vs. Absent)	0.582	0.125	0.163 (0.051-0.527)	0.562
	Serum Tumor Markers (Positive vs. Negative)	1.567	0.513	4.792 (2.456-10.401)	<0.001
	Serum Calcium (per 0.1 mmol/L increase)	1.093	0.267	2.443 (1.532-4.897)	<0.001
	Serum ALP (per 10 U/L increase, centered)	1.117	0.189	3.177 (1.172-6.399)	<0.001
	MRI Soft Tissue Mass (Present vs. Absent)	1.174	0.332	3.652 (2.218-9.603)	<0.001
	MRI Vertebral Compression Fracture (Present vs. Absent)	0.205	0.198	0.105 (0.022-0.429)	0.896
Radiomics Features	T1WI - Surface Area/Volume Ratio	2.292	0.576	6.635 (2.341-18.812)	<0.001
	T1WI - GLCM Entropy	1.194	0.489	5.218 (2.013-12.764)	<0.001
	T2WI - Major Axis/Minor Axis Ratio	1.137	0.614	3.062 (1.685-7.447)	<0.001
	T2WI - Short-Run Low Gray-Level Emphasis	0.458	0.232	0.293 (0.036-1.045)	0.763
	FS-T2WI - Gray-Level Non-Uniformity	2.315	0.598	5.742 (3.487-13.506)	<0.001
	FS-T2WI - Small Area High Gray-Level Emphasis	1.086	0.385	0.475 (0.053-1.139)	0.602
	Wavelet - db4 Mid-Frequency Entropy	1.753	0.524	2.768 (1.265-5.683)	<0.001

VAS, Visual Analogue Scale; ALP, Alkaline Phosphatase; MRI, Magnetic Resonance Imaging; GLCM, Gray Level Co-occurrence Matrix; CI, Confidence Interval.

This table presents the regression coefficients of all candidate clinical variables and the top 7 most discriminative radiomics features. The complete set of 11 radiomics features selected by LASSO regression (**Table 3**) was used to construct the integrated Rad-score, which was the only radiomics variable entered into the final multinomial logistic regression model. Variables with P < 0.05 were retained as independent predictors. The final model included five significant clinical variables and the integrated Rad-score. The reference category for the multinomial logistic regression model is the Primary Benign Group.

### 3.4 Development and performance of the radiomics nomogram

**Figure 4:**
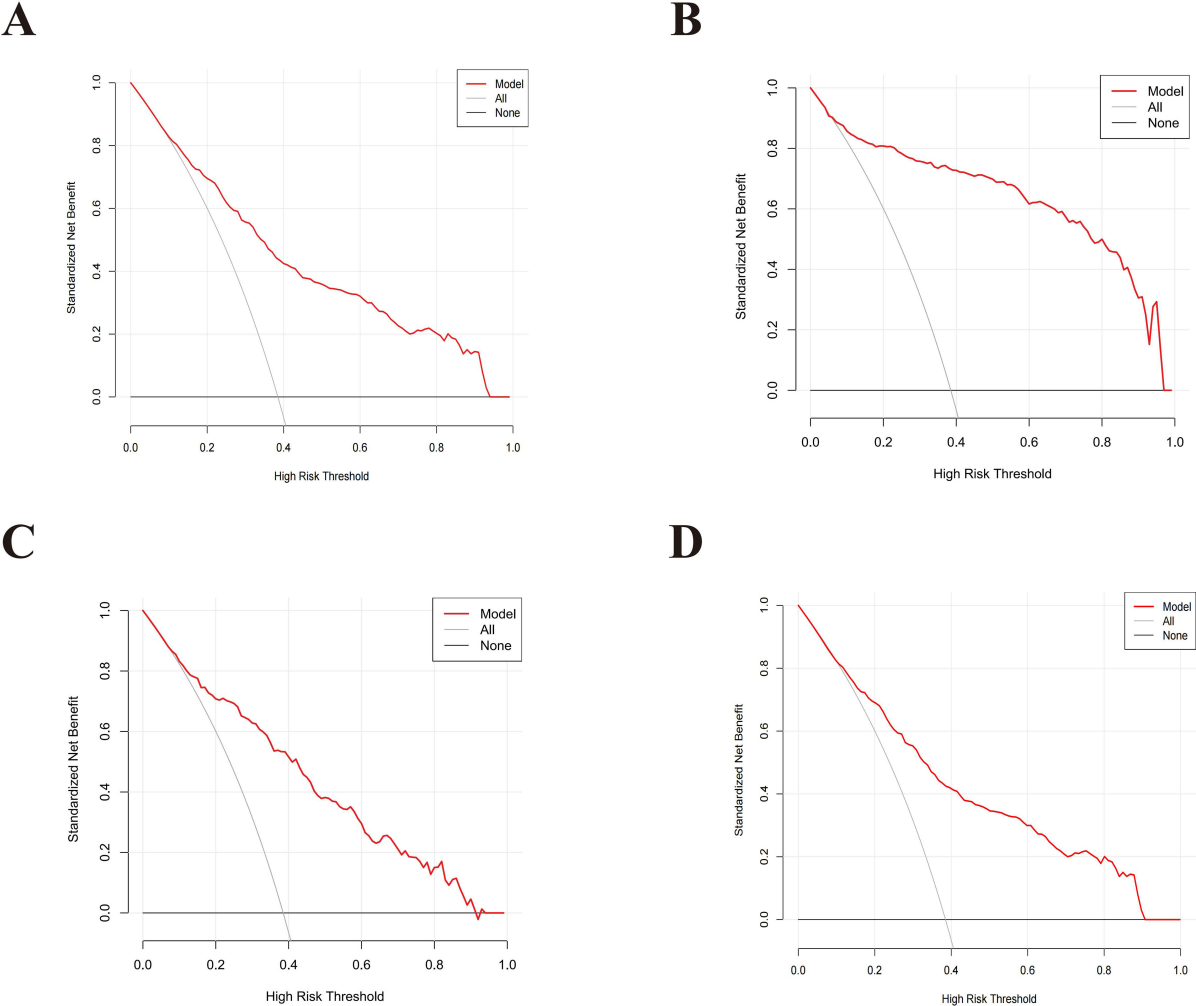
Decision curve analysis (DCA) evaluating the clinical utility of the nomogram in the independent test set (n=30). **(A)** Macro-average DCA curve summarizing the overall clinical utility of the three-class model. **(B)** One-vs-rest DCA curve for metastatic tumors (metastatic vs. primary benign + primary malignant). **(C)** One-vs-rest DCA curve for primary benign tumors (primary benign vs. primary malignant + metastatic). **(D)** One-vs-rest DCA curve for primary malignant tumors (primary malignant vs. primary benign + metastatic).

The six final predictors (five clinical variables and one integrated Rad-score) were integrated to construct a radiomics nomogram that generates individual predicted probabilities for each of the three tumor types (**Figure 1**). The nomogram demonstrated acceptable discriminatory performance. In the training set, the macro-average AUC was 0.921 (95% CI: 0.897–0.945). In the independent test set, the macro-average AUC was 0.887 (95% CI: 0.832–0.931) (**Figure 2A**). For one-vs-rest analysis in the test set, the AUCs were 0.892 (95% CI: 0.835–0.939) for primary benign tumors, 0.856 (95% CI: 0.789–0.912) for primary malignant tumors, and 0.904 (95% CI: 0.847–0.948) for metastatic tumors (**Figure 2B**). For pairwise discrimination, the AUCs were 0.912 (95% CI: 0.856–0.957) for metastatic vs. primary benign, 0.865 (95% CI: 0.798–0.919) for primary malignant vs. primary benign, and 0.834 (95% CI: 0.761–0.892) for primary malignant vs. metastatic. These results indicate that the nomogram performs best in identifying metastatic tumors, while discrimination of primary malignant tumors is relatively more challenging. This is consistent with clinical practice, as primary malignant spinal tumors often share overlapping imaging features with both benign lesions and metastases. Importantly, despite this relative challenge, the model still maintains sufficient statistical discriminatory power for identifying primary malignant tumors, with an one-vs-rest AUC of 0.856 and pairwise AUCs exceeding 0.83. Detailed classification performance metrics (sensitivity, specificity, positive predictive value, negative predictive value, and F1-score) for each tumor type at the default 0.50 threshold are provided in **Supplementary Table 3**. Calibration analysis in the test set showed good agreement between predicted probabilities and actual outcomes (Hosmer-Lemeshow test: χ² = 7.213, P = 0.407; **Figure 3**), with a mean absolute error of <5%. Decision curve analysis demonstrated that the nomogram provided a superior standardized net benefit compared to the “treat-all” or “treat-none” strategies across the clinically relevant threshold range of 0.10–0.94 (**Figure 4A**). In the one-vs-rest analysis, the nomogram maintained superior net benefit for all three tumor types across their respective clinically relevant threshold ranges: For metastatic tumors: Superior net benefit across thresholds of 0.08–0.95 (**Figure 4B**); For primary benign tumors: Superior net benefit across thresholds of 0.10–0.92 (**Figure 4C**); For primary malignant tumors: Superior net benefit across thresholds of 0.15–0.88 (**Figure 4D**). At the commonly used threshold probability of 0.50, based on the independent test set results extrapolated to a hypothetical cohort of 100 patients, utilization of the nomogram could potentially prevent approximately 18 unnecessary invasive procedures and 15 missed malignant diagnoses.

## 4 Discussion

The accurate preoperative discrimination of lumbar spinal tumor types remains a significant clinical challenge, directly impacting subsequent therapeutic pathways (20). In this study, we developed and validated a multiparametric MRI-based radiomics nomogram that combines radiomic features and key clinical predictors. The model demonstrated acceptable performance in differentiating among primary benign, primary malignant, and metastatic tumors, offering a promising non-invasive tool to augment clinical decision-making.

Our integrated nomogram achieved a macro-average AUC of 0.887 (95% CI: 0.832–0.931) in the independent test set, indicating acceptable overall discriminatory performance. Notably, it performed best in distinguishing metastatic from primary benign tumors (AUC = 0.912, 95% CI: 0.856–0.957), a clinically critical distinction as it often dictates a shift from localized surgical management to systemic therapy and palliative care (21). The model’s performance across all binary comparisons supports its utility in the complex diagnostic landscape of lumbar spinal tumors, where overlapping conventional imaging features are common.

A notable observation from the nomogram is that the highest probability of primary malignant tumors occurs within a relatively narrow total points interval of 150–180. This phenomenon is primarily attributed to three factors: Inherent biological characteristics: Primary malignant spinal tumors represent an intermediate biological entity, sharing overlapping clinical and radiological features with both benign lesions (relatively indolent growth in early stages) and metastatic tumors (invasive growth and bone destruction in advanced stages). This inherent intermediate nature results in a narrow transition zone in the probability distribution. Epidemiological sample size distribution: The sample size of primary malignant tumors (n=22) was smaller than that of benign (n=30) and metastatic (n=48) tumors in our cohort, which reflects the real-world epidemiological pattern where primary spinal malignancies are relatively rare. Intrinsic limitation of linear nomogram visualization: Traditional linear nomograms use a uniform scale to display non-linear probability relationships, which inherently leads to reduced resolution in regions where multiple probability curves intersect and change rapidly.

The good calibration of the nomogram, as evidenced by the Hosmer-Lemeshow test (P = 0.407) and the calibration curve, indicates that the predicted probabilities reliably reflect the actual likelihood of tumor type. This is crucial for clinical application, as it builds clinician trust in the model’s output. Furthermore, the Decision Curve Analysis confirmed the model’s superior net benefit across a wide, clinically relevant threshold probability range. This signifies that using our nomogram to guide decisions—such as whether to proceed with biopsy, definitive surgery, or further systemic staging—would lead to better patient outcomes compared to default strategies of intervening on all or no patients (22).

The core of our radiomics signature comprised 11 features derived from standard clinical sequences. The biological plausibility of these features enhances the interpretability of our model. For instance, a higher “Surface Area/Volume Ratio” from T1WI, indicative of a more irregular and spiculated tumor border, was strongly associated with malignancy, aligning with known pathological behaviors of invasive tumors (23). Similarly, the prominence of “Gray-Level Non-Uniformity” from FS-T2WI in the signature likely reflects the marked intratumoral heterogeneity characteristic of metastatic deposits (24). The fact that FS-T2WI contributed the most features (4 out of 11) underscores its value in lumbar tumor assessment, potentially due to its superior sensitivity in depicting bone marrow edema and soft tissue components, which are often pivotal for diagnosis.

The integration of these quantitative imaging traits with five readily available clinical variables (VAS score, serum tumor markers, calcium, ALP, and soft tissue mass on MRI) created a model superior to either dataset alone. This synergy mirrors the actual clinical diagnostic process, where imaging findings are always interpreted in the context of the patient’s clinical presentation. For example, while a soft tissue mass on MRI is a recognized indicator of aggression (25), its predictive power is substantially augmented when combined with elevated serum markers and calcium levels, creating a more holistic profile of the disease.

Previous radiomics studies in spinal tumors have often focused on binary classification, such as distinguishing benign from malignant lesions or identifying specific tumor types (26). While valuable, this approach does not fully address the tripartite differential diagnosis that clinicians face. Our study directly addresses this gap by constructing a three-class prediction model, which is more aligned with clinical reasoning and decision trees. A few studies have attempted multi-class classification but often relied on a single MRI sequence or did not integrate clinical data (27). The strength of our work lies in the use of multiparametric MRI and the construction of a clinically translatable nomogram that provides individualized risk probabilities, a feature highly valued in personalized medicine.

In daily clinical practice, this nomogram may support decision-making at three key points. For patients with predicted benign probability >0.85 and mild symptoms, it may help avoid unnecessary biopsy and guide conservative management with 3-month imaging follow-up. For patients with predicted metastatic probability >0.85, it may accelerate systemic staging and multidisciplinary oncology evaluation. For patients with predicted primary malignant probability >0.80, it may assist surgeons in planning appropriate surgical margins. Intermediate-probability cases (0.40–0.85) still require biopsy for definitive diagnosis.

The proposed radiomics nomogram represents a preliminary non-invasive decision-support tool for the tripartite differentiation of primary benign, primary malignant, and metastatic lumbar spinal tumors. However, its widespread clinical implementation is currently limited by the study’s methodological constraints, and further prospective multicenter external validation using data acquired from different MRI scanners, vendors, and standardized imaging protocols is essential before routine clinical use. Several limitations of our study should be acknowledged. First, the retrospective design may introduce selection bias, and all MRI examinations were completed on the same GE 3.0T scanner with a unified standardized protocol. This intentional design minimized technical variability and established a high-performance benchmark model, but it also significantly limits the generalizability of the model to clinical settings using different MRI equipment, field strengths, or imaging protocols. The current model is only applicable to images collected with similar equipment and protocols, which restricts its immediate clinical utility across diverse healthcare institutions. Second, while our feature extraction and selection pipeline was systematic and compliant with the IBSI guidelines, the radiomics workflow can be influenced by multiple factors, including segmentation variability and software differences. To mitigate this, we performed systematic inter- and intra-observer reproducibility assessments, and only features with ICC ≥ 0.85 were retained for model construction. Third, the highest probability of primary malignant tumors occurs within a relatively narrow interval (150–180 total points) in the nomogram, which may result in reduced resolution for manual visual evaluation. To address this limitation, a detailed probability lookup table for this critical interval is provided in **Supplementary Table 2** to facilitate accurate clinical evaluation. Clinicians are also advised to combine the nomogram results with other clinical information for comprehensive judgment. Fourth, for machine learning research, the total sample size of this study is relatively small, especially considering that primary malignant spinal tumors are inherently rare. Notably, only 7 cases of primary malignant tumors were included in the independent test set, which may reduce the statistical power of subgroup performance assessment and limit the reliability of the model’s predictions for this specific tumor type. Although mitigation measures such as 10-fold cross-validation and stratified sampling were adopted, the small sample size still inherently increases the risk of overfitting and restricts the nomogram’s clinical applicability. Fifth, we adopted stratified random sampling for cohort division rather than center-based external validation. Although stratified random sampling ensured balanced distribution of tumor types between the training and test sets, it does not provide the same level of evidence for generalizability as independent center-based external validation, which is a critical requirement for clinical translation. This limitation further restricts the current nomogram’s usability in routine clinical practice. Future prospective multi-center studies with larger and more evenly distributed cohorts will conduct center-based external validation to further confirm the model’s generalizability across different clinical settings. Sixth, this study did not compare the model’s performance with that of radiologists with different levels of experience. This is an important limitation, as head-to-head comparison with human experts is needed to evaluate the true added clinical value of the radiomics model. A multi-reader multi-case (MRMC) study is planned to assess the model’s impact on diagnostic accuracy, inter-observer agreement, and diagnostic time. Finally, we focused on the most common clinical MRI sequences. Incorporating advanced sequences such as diffusion-weighted imaging (DWI) or dynamic contrast-enhanced (DCE) MRI could provide additional biological information and further improve the model’s discriminatory performance.

## 5 Conclusion

In conclusion, we have successfully developed and validated a multiparametric MRI-based radiomics-clinic nomogram that effectively preoperatively discriminates among primary benign, primary malignant, and metastatic lumbar spinal tumors. This tool shows acceptable discrimination, good calibration, and preliminary clinical utility, and represents a promising non-invasive decision-making auxiliary tool that requires further multicenter validation before widespread clinical implementation.

## Data Availability

The original contributions presented in the study are included in the article/**Supplementary Material**. Further inquiries can be directed to the corresponding author.
