# Supplementary material for: Development and validation of a multiparametric MRI-based radiomics nomogram for the tripartite discrimination of primary benign, primary malignant, and metastatic lumbar spinal tumors
Source: Front Oncol. 2026 Jun 10;16:1772338. doi: 10.3389/fonc.2026.1772338 (PMC13290590; doi:10.3389/fonc.2026.1772338)
Supplement: Supplementary file 1 [file Table1.docx]

**Supplementary Table 1. Comparison of Baseline Characteristics Between the Training Set and the Independent Test Set.**

| **Characteristic** | **Training Set**  **(n=70)** | **Independent Test Set**  **(n=30)** | **Statistic** | **P value** |
| --- | --- | --- | --- | --- |
| **Demographics and Symptoms** |  |  |  |  |
| Age (years) | 51.2 ± 11.3 | 50.7 ± 10.8 | t=0.214 | 0.831 |
| Gender, n(%) |  |  | χ²=0.048 | 0.826 |
| Male | 38 (54.3) | 17 (56.7) |  |  |
| Female | 32 (45.7) | 13 (43.3) |  |  |
| BMI (kg/m²) | 23.4 ± 2.3 | 23.2 ± 2.1 | t=0.417 | 0.678 |
| Smoking History, n(%) |  |  | χ²=0.010 | 0.921 |
| No | 52 (74.3) | 22 (73.3) |  |  |
| Yes | 18 (25.7) | 8 (26.7) |  |  |
| ECOG Score, n(%) |  |  | χ²=0.014 | 0.905 |
| 0-1 | 59 (84.3) | 25 (83.3) |  |  |
| 2 | 11 (15.7) | 5 (16.7) |  |  |
| VAS Pain Score | 5.5 (3.0, 7.5) | 5.0 (2.5, 7.0) | Z=0.682 | 0.495 |
| **Signs and Serology** |  |  |  |  |
| Neurological Compression Symptoms, n(%) |  |  | χ²=0.093 | 0.760 |
| Present | 35 (50.0) | 16 (53.3) |  |  |
| Absent | 35 (50.0) | 14 (46.7) |  |  |
| Comorbidities, n(%) |  |  | χ²=0.086 | 0.994 |
| None | 41 (58.6) | 17 (56.7) |  |  |
| Hypertension | 16 (22.9) | 7 (23.3) |  |  |
| Diabetes Mellitus | 8 (11.4) | 4 (13.3) |  |  |
| Hypertension + Diabetes | 5 (7.1) | 2 (6.7) |  |  |
| Serum Tumor Markers, n(%) |  |  | χ²=0.017 | 0.896 |
| Negative | 34 (48.6) | 15 (50.0) |  |  |
| Positive | 36 (51.4) | 15 (50.0) |  |  |
| Serum Calcium (mmol/L) | 2.6 ± 0.3 | 2.5 ± 0.3 | t=0.529 | 0.598 |
| Serum ALP (U/L) | 118 ± 28 | 115 ± 26 | t=0.497 | 0.620 |
| **MRI Characteristics** |  |  |  |  |
| Vertebral Level Involved, n(%) |  |  | χ²=0.016 | 0.992 |
| L1-L2 | 11 (15.7) | 5 (16.7) |  |  |
| L3-L4 | 28 (40.0) | 12 (40.0) |  |  |
| L5-S1 | 31 (44.3) | 13 (43.3) |  |  |
| Time from Symptom Onset to MRI (months) | 3.0 ± 1.3 | 3.1 ± 1.2 | t=0.358 | 0.721 |
| MRI with Soft Tissue Mass, n(%) |  |  | χ²=0.048 | 0.826 |
| Yes | 39 (55.7) | 16 (53.3) |  |  |
| No | 31 (44.3) | 14 (46.7) |  |  |
| MRI with Vertebral Compression Fracture, n(%) |  |  | χ²=0.008 | 0.930 |
| Yes | 31 (44.3) | 13 (43.3) |  |  |
| No | 39 (55.7) | 17 (56.7) |  |  |
| **Tumor Type Distribution** |  |  | χ²=0.051 | 0.975 |
| Primary Benign | 21 (30.0) | 9 (30.0) |  |  |
| Primary Malignant | 15 (21.4) | 7 (23.3) |  |  |
| Metastatic | 34 (48.6) | 14 (46.7) |  |  |

BMI, Body Mass Index; ECOG, Eastern Cooperative Oncology Group; VAS, Visual Analogue Scale; ALP, Alkaline Phosphatase; MRI, Magnetic Resonance Imaging.
