# Supplementary material for: Development and validation of a multiparametric MRI-based radiomics nomogram for the tripartite discrimination of primary benign, primary malignant, and metastatic lumbar spinal tumors
Source: Front Oncol. 2026 Jun 10;16:1772338. doi: 10.3389/fonc.2026.1772338 (PMC13290590; doi:10.3389/fonc.2026.1772338)
Supplement: Supplementary file 2 [file Table2.docx]

**Supplementary Table 2. Probability Lookup Table for the Critical Interval (150–180 Total Points).**

| **Total Points** | **Primary Benign Probability** | **Primary Malignant Probability** | **Metastatic Probability** | **Highest Probability Class** |
| --- | --- | --- | --- | --- |
| 150 | 0.45 | 0.35 | 0.20 | Primary Benign |
| 155 | 0.42 | 0.36 | 0.22 | Primary Benign |
| 160 | 0.38 | 0.36 | 0.26 | Primary Benign |
| 165 | 0.35 | 0.37 | 0.28 | Primary Malignant |
| 170 | 0.32 | 0.37 | 0.31 | Primary Malignant |
| 175 | 0.28 | 0.38 | 0.34 | Primary Malignant |
| 180 | 0.25 | 0.38 | 0.37 | Primary Malignant |

Note: The probability distribution in the 150–180 interval reflects the intermediate biological nature of primary malignant tumors, which share features with both benign and metastatic lesions. For cases falling within this range, clinicians are strongly recommended to combine the nomogram results with other clinical information for comprehensive judgment.
