# Supplementary material for: Development and validation of a multiparametric MRI-based radiomics nomogram for the tripartite discrimination of primary benign, primary malignant, and metastatic lumbar spinal tumors
Source: Front Oncol. 2026 Jun 10;16:1772338. doi: 10.3389/fonc.2026.1772338 (PMC13290590; doi:10.3389/fonc.2026.1772338)
Supplement: Supplementary file 3 [file Table3.docx]

**Supplementary Table 3. Detailed Performance Metrics of the Three-Class Model in the Independent Test Set.**

| **Tumor Type** | **Sensitivity (95% CI)** | **Specificity (95% CI)** | **PPV**  **(95% CI)** | **NPV**  **(95% CI)** | **F1-Score** |
| --- | --- | --- | --- | --- | --- |
| Primary Benign | 0.889 (0.518–0.993) | 0.905 (0.696–0.982) | 0.800 (0.444–0.975) | 0.950 (0.751–0.999) | 0.842 |
| Primary Malignant | 0.714 (0.290–0.963) | 0.913 (0.720–0.989) | 0.714 (0.290–0.963) | 0.913 (0.720–0.989) | 0.714 |
| Metastatic | 0.929 (0.661–0.996) | 0.875 (0.617–0.971) | 0.867 (0.595–0.976) | 0.933 (0.681–0.998) | 0.897 |
| Macro-average | 0.844 | 0.898 | 0.794 | 0.932 | 0.818 |

Note: For the primary malignant class, the identical values of sensitivity and PPV, and specificity and NPV are a mathematical coincidence resulting from the equal number of false negatives (FN=2) and false positives (FP=2) in this small subgroup (n=7). The wide 95% confidence intervals reflect the statistical uncertainty associated with this limited sample size.
